# Over‐Representation of *TTN* Truncating Variants in a Finnish Cohort of Patients With Axial Myopathy

**DOI:** 10.1111/ene.70537

**Published:** 2026-02-17

**Authors:** Maria Francesca Di Feo, Giuliana Capece, Marco Savarese, Bjarne Udd, Manu Jokela, Johanna Palmio

**Affiliations:** ^1^ Folkhälsan Research Center Helsinki Finland; ^2^ University of Helsinki Helsinki Finland; ^3^ Department of Neuroscience, Rehabilitation, Ophthalmology, Genetics, and Maternal and Child Health (DINOGMI) University of Genoa Genova Italy; ^4^ Department of Neuroscience DNS University of Padova Padova Italy; ^5^ Neuromuscular Research Center Tampere University and University Hospital Tampere Finland; ^6^ Vasa Central Hospital Ostrobothnia Finland; ^7^ Clinical Neurosciences University of Turku Turku Finland; ^8^ Neurocenter Turku University Hospital Turku Finland

**Keywords:** axial myopathy, genetics, neuromuscular disorders, Titin, titinopathies

## Abstract

**Background:**

Axial myopathies present with late onset selective paravertebral weakness causing bent spine/camptocormia or dropped head, and the genetic basis remains currently only partially understood. Truncating variants in *TTN* (TTNtv) are found in about 1% of the general population and, when biallelic, cause recessive titinopathies. Also, TTNtv located in cardiac exons are known to confer an increased risk of cardiomyopathy, with incomplete penetrance.

**Methods:**

We retrospectively analyzed 55 Finnish adults with late‐onset axial myopathy evaluated at the Tampere Neuromuscular Center (2015–2025). Clinical, imaging, and histopathological data were collected, and genetic testing was performed using the MYOcap targeted next‐generation sequencing panel.

**Results:**

Heterozygous TTNtv were identified in 9 of 55 patients (16%), representing a significant enrichment compared with the general population (odds ratio = 14.1; *p* ≈5 × 10^−8^). The variants were ultra‐rare, distributed across different exons expressed in skeletal muscle, and five were absent from gnomAD. Mean age at onset was 60 ± 11 years; six patients were female, and five reported a positive family history. Camptocormia was the main presentation, with muscle MRI showing a consistent fatty‐fibrous replacement of paravertebral muscles in all cases. Muscle biopsies revealed either myopathic or myofibrillar changes without a uniform pattern.

**Conclusions:**

Heterozygous TTNtv are significantly enriched in patients with late‐onset axial myopathy, suggesting a potential contribution to this phenotype. These findings broaden the clinical spectrum of titin‐related diseases and support inclusion of *TTN* in genetic testing for idiopathic axial myopathies.

## 1 Introduction

Axial myopathies are characterized by weakness of the paravertebral muscles, which may be isolated or associated with some limb muscles' weakness [1].

Several genetic myopathies have an axial involvement with severe limb weakness presenting in very early ages with poor head control, then leading to severe forms of scoliosis and rigid spine due to contractures of paraspinal muscles. In later adulthood typical clinical presentations are bent spine/camptocormia (i.e., forward trunk flexion relieved in the supine position.) or dropped head [1].

Myopathies due to pathogenic variants in *SEPN1* and *LMNA* are characterized by a distinctive severe early involvement of paravertebral muscles, whereas pathogenic variants in *RYR1* may cause a later onset myopathy with lumbar hyperlordosis and cervical camptocormia [1, 2, 3]. Among the metabolic disorders, late‐onset Pompe disease patients may present an isolated bent spine [1]. Finally, a heterogeneous group of myopathies causes paraspinal weakness in the context of a more widespread muscle weakness, such as facioscapulohumeral muscular dystrophy and myotonic dystrophy type I [1].

Although some pathophysiological mechanisms have been identified (e.g., the vulnerability of axial muscles to oxidative stress), the precise factors underlying the distinctive paravertebral weakness observed in certain myopathies remain unknown [2]. Although approximately 50% of camptocormia cases fall within the clinical category of neuromuscular and musculoskeletal disorders, only a small proportion reach a definitive molecular diagnosis [4]. Notably, neurodegenerative disorders—especially idiopathic Parkinson's disease—should be included in the differential diagnosis in adult patients [4]. In most patients with axial myopathy, no causative mutation has been identified, while in other cases, genetic testing is not even performed [4]. Even the exact prevalence remains unknown, as most patients have been described in isolated case reports or small case series.

Interestingly, many patients with severe early recessive titinopathies show paravertebral involvement, often associated with variable degrees of contractures, scoliosis, and restrictive respiratory impairment, with a possible concomitant cardiomyopathy [5, 6].

Recently, fatty‐fibrous substitution in axial muscles has been observed in a cohort of 25 patients with heterozygous likely pathogenic/pathogenic *TTN* truncating variants (TTNtvs), in exons expressed both in heart and skeletal muscles [7].

Herein, we report a monocentric cohort of 55 Finnish patients with late‐onset axial myopathy presenting with camptocormia and/or dropped head.

## 2 Methods

We retrospectively reviewed our medical records to identify patients with axial myopathy evaluated at the Tampere Neuromuscular Center between 2015 and 2025. Axial myopathy was defined by selective paravertebral muscle weakness, clinically presenting as camptocormia (bent spine), and radiologically proven. Acquired neuromuscular disorders (i.e., autoimmune and endocrine diseases) were systematically excluded.

We recorded clinical data on disease onset, family history, and coexistence of cardiomyopathy. All patients also underwent a neurological examination. Genetic testing was performed using a targeted next‐generation sequencing (NGS) panel, MYOcap (File S1), previously described by Evilä et al., and data were analyzed with a standardized pipeline [8]. Variant classification was performed according to ACMG guidelines, and only variants classified as pathogenic or likely pathogenic were included in the analysis. Minor allele frequency (MAF) for each variant was taken from gnomAD v4.1.0.

Diagnostic work‐up also included muscle magnetic resonance imaging of the limb and paravertebral muscles using 1.5‐T magnetic resonance scanners (Siemens and Philips) and an open skeletal muscle biopsy with routine histological and histochemical analyses performed according to standard procedures.

## 3 Results

A total of 55 patients with axial myopathy were identified, and all of them underwent genetic testing. In nine of these cases (16%), a heterozygous TTNtv classified as likely pathogenic or pathogenic (LP/P) according to ACMG criteria was detected (Table 1). No additional LP/P variants that could account for the observed phenotype were detected. Among the nine identified variants, seven are unique (two reoccur). The variants are distributed throughout the gene, in exons with variable PSI values but all expressed in skeletal muscle, with no apparent clustering (Figure S1) [9]. All variants are unpublished and extremely rare; four are reported in gnomAD v4.1.0, while three are absent (Table S1). The highest minor allele frequency was observed for the c.1455dup p.(Ala486SerfsTer26) variant (P1), with a global MAF of 3.1 × 10^−6^ and a Finnish MAF of 7.8 × 10^−5^, corresponding under Hardy–Weinberg equilibrium to an expected heterozygous carrier frequency of approximately 1 in 6400 in the Finnish population.

**TABLE 1:** Clinical and genetic features of the patients.

PT	Sex	Age at onset (y)	Axial weakness	Widespread muscle weakness	Loss of ambu‐lation	Cardiomyopathy	FFR in axial muscles at MRI	Muscle biopsy	Site muscle biopsy	Histological pattern	Family members with camptocormia	TTNtv	TTN region	Exon	Adult skeletal PSI	Adult cardiac PSI
1	F	47	Camptocormia	No	No	No	Yes	Yes	Vastus lateralis	Mild myopathic pattern	Yes (father, not analysed)	c.1455dup p.(Ala486SerfsTer26)	Z‐disk	9	95%	99%
2	M	75	Camptocormia	No	No	No	Yes	Yes	Tibialis anterior	Myofibrillar myopathy	No	c.3531T>A, p.(Tyr1177Ter)	near Z‐disk	22	98%	99%
3	M	60	Camptocormia	No	No	No	Yes	Yes	Thoracic paraspinal	End‐stage myopathy	No	c.28103del, p.(Ala9368GlufsTer12)	I‐band	98	94%	51%
4	F	55	Camptocormia	Yes, proximal UL/LL	No	No	Yes, +FFR in LL	Yes	Lumbar paraspinal (1), vastus lateralis (2)	End‐stage myopathy (1), myopathic pattern (2)	Yes (pt 5)	c.30531del, p.(Lys10177AsnfsTer57)	I‐band	110	85%	30%
5	F	73	Camptocormia	No	No	No	Yes	Yes	Lumbar paraspinal	Myofibrillar myopathy	Yes (pt 4)	c.30531del, p.(Lys10177AsnfsTer57)	I‐band	110	85%	30%
6	F	NA	Bent spine only with activity/walking	No	No	No	Yes	No	No	No	Yes (pt 7)	c.39492dup, p.(Glu13165Ter)	I‐band	208	56%	11%
7	F	50	Bent spine only with activity/walking	No	No	No	Yes	Yes	Paraspinal	End‐stage myopathy	Yes (pt 6)	c.39492dup, p.(Glu13165Ter)	I‐band	208	56%	11%
8	F	70	Camptocormia	No	No	No	yes, +FFR in LL	Yes	Thoracic paraspinal	Mitochondrial abnormalities+myofibrillary pattern	NA	c.48446dup, p.(Met16149IlefsTer9)	A‐band	259	90%	94%
9	M	50	Camptocormia	No	No	Asymptomatic mildly reduced EF	Yes	Yes	deltoid	Myofibrillar myopathy	No	c.62733G>A, p.(Trp20911Ter)	A‐band	305	91%	95%

*Note:* All variants are annotated according to the canonical *TTN* transcript (NM_001267550.2). Titin regions are reported based on Cardiodb (https://www.cardiodb.org/titin/titin_transcripts.php). Exon numbering follows the LRG system (also adopted by LOVD), which is recommended for clinical reporting. Exon usage data (PSI values) are taken from Di Feo et al., 2024, and are also available at (http://psivis.it.helsinki.fi:3838/TTN_PSIVIS/).

Abbreviations: EF, ejection fraction; F, female; FFR, fatty‐fibrous replacement; LL, lower limbs; M, male; NA, information not available; PT, patient; UL, upper limbs; y, years.

Our cohort included 6 females and 3 males. The mean age at onset was 60 ± 11.3 years (range 47–75). A positive family history of camptocormia was present in five patients (55.6%), including two pairs of sisters; one additional patient reported an affected father (not genetically tested). All patients exhibited camptocormia. Neurological examination revealed isolated paravertebral weakness in all patients, except for a single case with also proximal limb weakness. No patient was wheelchair‐bound, although two patients needed walking aids for long‐distance walking.

Four patients carried a TTNtv in an exon with high cardiac expression (Percentage Spliced In > 90%), but no patient was affected by cardiomyopathy, with only one case (P9) showing an asymptomatic mild reduction of ejection fraction.

**FIGURE 1:**
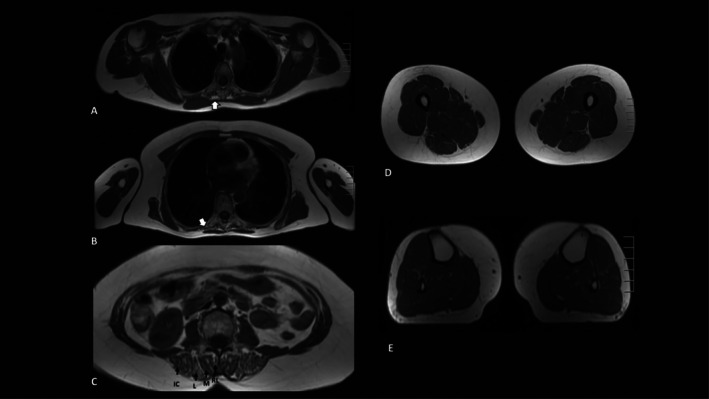
Muscle MRI findings in patient 6. Axial T1‐weighted images show selective involvement of the paravertebral muscles at the thoracic (A, B) and lumbar levels (C) with no significant abnormalities in the shoulder girdle (A), upper limbs (B), thighs (D), and legs (E). Thoracic sections demonstrate fibrous fatty replacement (FFR) and atrophy within the transversospinalis and erector spinae muscle groups (A and B, white arrow). Lumbar section (C) shows diffuse and patchy FFR of the paravertebral muscles. IC, iliocostal; L, longissimus; M, multifidus; RL, rotatores lumborum.

Muscle MRI was performed in all patients, revealing fatty‐fibrous replacement (FFR) in the paravertebral muscles (Figure 1; Table 1). In two cases, signs of FFR were also found in lower limbs, with concomitant muscle weakness in one.

Eight patients underwent muscle biopsy: paraspinal muscles in four patients, both paraspinal and vastus lateralis in a single case, and limb muscles in one case each. A myofibrillar pattern was observed in four cases, with mitochondrial abnormalities also observed in a single case. In the remaining cases, a myopathic pattern was observed, mostly in the end stage (3/4).

## 4 Discussion

Clinical findings in our cohort were consistent with previous descriptions of axial myopathy, showing a late onset, female predominance, and frequent familial aggregation [10, 11]. Camptocormia/bent spine represented the main presentation, with weakness largely confined to paravertebral muscles. Muscle MRI and biopsies confirmed characteristic FFR and myopathic changes, consistent with published data. Along with an increase in connective tissue and adipose infiltration, myopathic changes have also been previously reported in axial myopathy [11, 12]. Myofibrillar disarray appeared more frequent in our cohort than previously reported and may reflect alterations in sarcomere structure potentially influenced by *TTN* variants. However, the diagnostic value of muscle biopsy in axial myopathies remains debated, as paraspinal biopsies are rarely performed and normative reference data are lacking [1]. Histopathological interpretation is further challenged by the heterogeneity of axial muscle groups and by age‐related changes [1, 13, 14].

The prevalence of heterozygous TTNtv in the general population, without restricting to cardiac exons, is estimated to be up to 1% [13]. According to the analysis performed in 2015 by Akinrinade and colleagues, the prevalence of heterozygous TTNtv in the ExAC cohort was 813 out of 60,706 individuals (1.34%), when not restricted to cardiac exons. In our axial myopathy cohort, 9 out of 55 individuals (16%) carried a heterozygous TTNtv, representing a significantly higher prevalence (odds ratio = 14.1, 95% CI: 6.8–29.4; Fisher's exact test, *p* ≈5 × 10^−8^). These findings suggest an association between heterozygous *TTN* pathogenic variants and late‐onset axial myopathy, while not implying direct causality. Interestingly, independent evidence comes from a recent Italian cohort of 20 patients with axial myopathy, which reported a very similar prevalence of heterozygous TTNtv classified as LP/P: 3 out of 20 (15%) [15]. However, given that both studies are based on tertiary referral cohorts enriched for suspected hereditary neuromuscular disorders, the observed prevalence may not be directly generalizable to unselected clinical populations.

It must be underlined that if all TTNtv invariably led to late‐onset axial myopathy, the prevalence of this condition would be expected to be far higher than observed. The number of reported cases remains too limited to determine whether recurrent, high‐penetrance variants exist that might indicate preferential involvement of specific *TTN* regions or exons. Paraspinal muscles remain poorly studied, partly due to their limited accessibility, and it is not yet clear whether the titin isoforms expressed are the same as in other muscles or which exons are specifically included. However, evidence presented here, from both the literature and our cohort, suggests that multiple variants in different regions of the gene may be involved. This enrichment resembles what has been described for dilated cardiomyopathy associated with TTNtv, which are more common in individuals with cardiomyopathy than in healthy controls, but clearly show reduce penetrance [16]. In human genetics, low penetrance usually indicates the involvement of additional factors, which may include variants of uncertain pathogenicity *in trans* (e.g., missense variants, non‐coding variants in regulatory regions, etc.), variants in other genes (digenic or oligogenic mechanisms), and/or external modifiers such as medication use, comorbidities, lifestyle, and other environmental factors [17]. Taken together, these findings support the association of TTNtv with late‐onset axial myopathies, but they do not exclude the contribution of other determining factors—for example, almost all *TTN* missense variants remain of uncertain significance, and the presence of additional hits in trans in these patients cannot be ruled out. We recommend that molecular testing (NGS panels, exome, or genome sequencing) should be systematically performed in cases of idiopathic axial myopathy, and that *TTN* variants classified as LP/P be reported, and that even variants of uncertain significance may be considered for reporting at the laboratory's discretion. Systematic sharing of such findings will enable the study of larger cohorts, allowing the identification of additional genetic contributors and clarification of the underlying pathogenic mechanisms.

## Author Contributions

Study conception and design: M.J., B.U., M.S., M.F.D.F., J.P. Clinical data collection: M.J., J.P., B.U. Data analysis and interpretation: M.F.D.F., G.C., M.J., B.U., J.P., M.S. Writing‐original draft: M.F.D.F. and G.C. Writing‐review and editing: all co‐authors. All authors read, critically revised, and approved the final manuscript.

## Funding

This study was funded by the European Commission under the HORIZON EUROPE Framework Programme (grant #101080874 to MS), the Research Council of Finland (grants #339437, #346209, and #361979 to MS), Samfundet Folkhälsan (to MS and BU), the Sigrid Juselius Foundation (grant #230217 to MS and BU), the Finnish Cultural Foundation (Suomen Kulttuurirahasto, to MFDF), and the State funding for university‐level health research, Tampere University Hospital, Wellbeing services county of Pirkanmaa (Project number T67774 to JP).

## Disclosure

The authors have nothing to report.

## Ethics Statement

The study was performed in accordance with the Declaration of Helsinki, and all the patients provided written informed consent. The study was approved by the Ethics Review Board of Helsinki and Uusimaa Hospital District (number HUS/16896/2022) and by the Tampere Regional Ethics Committee (TRE), approval number R01185.

## Conflicts of Interest

The authors declare no conflicts of interest.

## Supporting information


**Figure S1:** Schematic representation of the location of *TTN* variants identified in the cohort along the titin protein, showing their distribution across the Z‐disk, I‐band, A‐band, and M‐line regions. Exon numbers corresponding to the affected regions are indicated.

**File S1:** Description of the MYOcap targeted next‐generation sequencing (NGS) panel. The panel includes all *TTN* coding exons and untranslated regions, as well as 180 genes known to be associated with myopathic phenotypes or considered candidate genes.

**Table S1:** ene70537‐sup‐0003‐TableS1.docx. *TTN* truncating variants identified in the study cohort. For each variant, genomic and protein annotation, global and Finnish (gnomAD v4.1.0) minor allele frequencies, affected isoforms, exon number, sarcomeric region, and protein domain are reported.

## Data Availability

The data that support the findings of this study are available from the corresponding author upon reasonable request.
