# Supplementary material for: Over‐Representation of TTN Truncating Variants in a Finnish Cohort of Patients With Axial Myopathy
Source: Eur J Neurol. 2026 Feb 17;33(2):e70537. doi: 10.1111/ene.70537 (PMC12911116; doi:10.1111/ene.70537)
Supplement: Supplementary file 1 — Figure S1: Schematic representation of the location of TTN variants identified in the cohort along the titin protein, showing their distribution across the Z‐disk, I‐band, A‐band, and M‐line regions. Exon numbers corresponding to the affected regions are indicated. [file ENE-33-e70537-s002.docx]

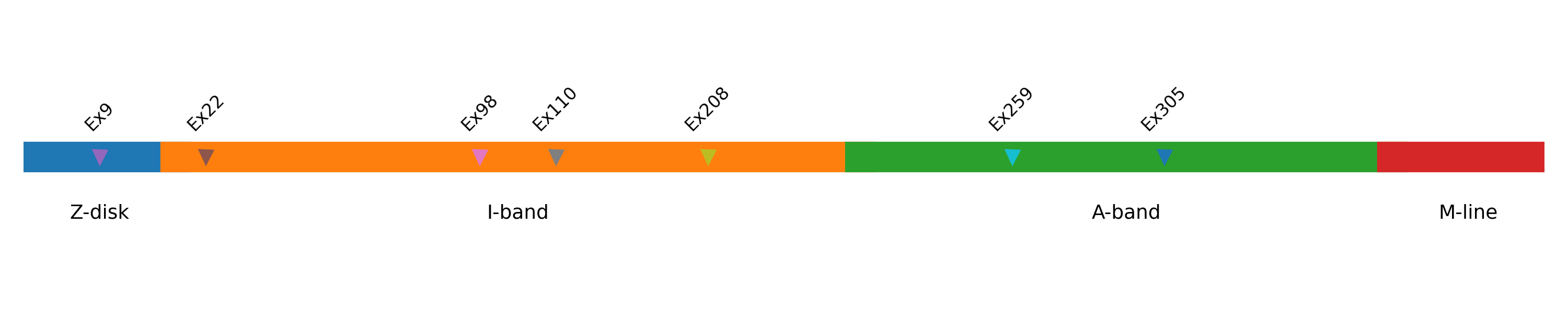


**Supplementary Figure 1.** Schematic representation of the location of TTN variants identified in the cohort along the titin protein, showing their distribution across the Z-disk, I-band, A-band, and M-line regions. Exon numbers corresponding to the affected regions are indicated.
