# Supplementary material for: Over‐Representation of TTN Truncating Variants in a Finnish Cohort of Patients With Axial Myopathy
Source: Eur J Neurol. 2026 Feb 17;33(2):e70537. doi: 10.1111/ene.70537 (PMC12911116; doi:10.1111/ene.70537)
Supplement: Supplementary file 2 — File S1: Description of the MYOcap targeted next‐generation sequencing (NGS) panel. The panel includes all TTN coding exons and untranslated regions, as well as 180 genes known to be associated with myopathic phenotypes or considered candidate genes. [file ENE-33-e70537-s003.docx]

**Supplementary File 1**. The MYOcap targeted NGS panel, as described by Evilä et al. (2016), has been routinely applied since 2014 in our neuromuscular research laboratory in Helsinki and at the Tampere Neuromuscular Center, Tampere University Hospital, achieving a mean sequencing depth of ~130× with ~95% of target bases covered at ≥20×. All *TTN* coding exons and UTRs were included, with overall coverage sufficient for reliable detection of single-nucleotide variants and small indels. The list of genes included in the MYOcap panel (180 known myopathic genes and candidate genes for muscle disease) is provided below.

*ABHD5, ACADS, ACADVL, ACTA1, ACTN3, ACVR1, AGL, ANO5, ATP2A1, B3GNT1, BAG3, BIN1, CACNA1A, CACNA1S, CAPN3, CAV3, CELF1, CFL2, CHKB, CKM, CLCN1, CMYA5, CNBP, CNTN1, COL6A1, COL6A2, COL6A3, CPT2, CRYAB, CSRP3, DAG1, DES, DMD, DMPK, DNAJB6, DNM2, DPM2, DPM3, DUX4, DYSF, EMD, ENO3, ETFA, ETFB, ETFDH, FBXO32, FHL1, FHL2, FKRP, FKTN, FLNC, GAA, GBE1, GNE, GYS1, ISCU, ISPD, ITGA7, KBTBD13, KCNE1, KCNE3, KCNJ2, KCNQ1, KLHL9, KY, LAMA2, LAMP1, LAMP2, LARGE, LDB3, LDHA, LDHB, LIFR, LMNA, MBNL1, MBNL2, MBNL3, MATR3, MEGF10, MSTN, MTM1, MTMR14, MYBPC2, MYBPC3, MYH1, MYH2, MYH3, MYH4, MYH7, MYH8, MYL1, MYL2, MYL3, MYL4, MYL5, MYL6, MYL6B, MYL7, MYL9, MYL10, MYL12A, MYL12B, MYLK, MYLK2, MYLK3, MYLK4, MYLIP, MYLPF, MYOM1, MYOM2, MYOM3, MYOT, MYOZ1, MYOZ2, MYOZ3, MYPN, NEB, NEBL, NBR1, NTRK1, OBSCN, OBSL1, PABPN1, PDLIM3, PDLIM5, PDLIM7, PFKM, PGAM2, PGK1, PGM1, PLEC, PLEKHG4, PLN, PNPLA2, POMGNT1, POMGNT2, POMT1, POMT2, PRKAG2, PTRF, PYGM, QDPR, RYR1, SCN4A, SEPN1, SGCA, SGCB, SGCD, SGCG, SLC22A5, SLC25A20, SOX10, SQSTM1, SRF, SYNE1, SYNE2, SYNE3, SYNPO2, TCAP, TIA1, TMEM5, TMEM43, TMOD3, TNNC1, TNNC2, TNNI1, TNNI2, TNNI3, TNNT1, TNNT3, TPM1, TPM2, TPM3, TRIM32, TRIM55, TRIM63, TTR, TTN, VCP, VMA21*
