# Supplementary material for: Over‐Representation of TTN Truncating Variants in a Finnish Cohort of Patients With Axial Myopathy
Source: Eur J Neurol. 2026 Feb 17;33(2):e70537. doi: 10.1111/ene.70537 (PMC12911116; doi:10.1111/ene.70537)
Supplement: Supplementary file 3 — Table S1: ene70537‐sup‐0003‐TableS1.docx. TTN truncating variants identified in the study cohort. For each variant, genomic and protein annotation, global and Finnish (gnomAD v4.1.0) minor allele frequencies, affected isoforms, exon number, sarcomeric region, and protein domain are reported. [file ENE-33-e70537-s001.docx]

| **Pt** | **Variant** | **GnomAD**  **MAF (global)** | **GnomAD  MAF (European Finnish)** | **Isoform** | **Exon** | **Region** | **Domain** |
| --- | --- | --- | --- | --- | --- | --- | --- |
| 1 | c.1455dup, p.(Ala486SerfsTer26) | 0.000003098 | 0.00007813 | All isoforms | Exon 9 | Z-disk | Z-repeat 2 |
| 2 | c.3531T>A, p.(Tyr1177Ter) | not found | not found | All isoforms | Exon 21 | I-band | Ig-4 |
| 3 | c.28103del, p.(Ala9368GlufsTer12) | not found | not found | N2A and N2BA only | Exon 98 | I-band | Ig-73 |
| 4,5 (*) | c.30531del, p.(Lys10177AsnfsTer57) | 0.0000006209 | 0.00001566 | N2A and N2BA only | Exon 110 | I-band | PEVK |
| 6,7 (*) | c.39492dup, p.(Glu13165Ter) | not found | not found | N2A and N2BA only | Exon 208 | I-band | PEVK |
| 8 | c.48446dup, p.(Met16149IlefsTer9) | not found | not found | All isoforms except Novex3 | Exon 259 | A-band | Fn III-4 |
| 9 | c.62733G>A, p.(Trp20911Ter) | 0.000001861 | 0.00001584 | All isoforms except Novex3 | Exon 305 | A-band | Fn III-39 |

**Supplementary Table 1**. *TTN* truncating variants identified in the study cohort. For each variant, genomic and protein annotation, global and Finnish (gnomAD v4.1.0) minor allele frequencies, affected isoforms, exon number, sarcomeric region, and protein domain are reported.

(*) The patients are sisters: see Table 1
